# Lockdown, Emotional Intelligence, Academic Engagement and Burnout in Pharmacy Students during the Quarantine

**DOI:** 10.3390/pharmacy8040194

**Published:** 2020-10-22

**Authors:** Jorge Moreno-Fernandez, Julio J. Ochoa, Inmaculada Lopez-Aliaga, Maria Jose M. Alferez, Manuel Gomez-Guzman, Sagrario Lopez-Ortega, Javier Diaz-Castro

**Affiliations:** 1 Department of Physiology, University of Granada, Campus de Cartuja s/n, 18071 Granada, Spain; jorgemf@ugr.es (J.M.-F.); jjoh@ugr.es (J.J.O.); milopez@ugr.es (I.L.-A.); malferez@ugr.es (M.J.M.A.); 2 Institute of Nutrition and Food Technology “José Mataix Verdú”, University of Granada, 18071 Granada, Spain; 3 Department of Pharmacology, University of Granada, 18071 Granada, Spain; mgguzman@ugr.es; 4 Gabinete Psicopedagógico, University of Granada, 18071 Granada, Spain; sagralo@ugr.es

**Keywords:** pharmacy students, lockdown, confinement, online teaching, emotional intelligence

## Abstract

The recent appearance and rapid spread of the new SARS-CoV-2 coronavirus meant taking unprecedented measures to control the pandemic, which in Spain forced a state of alarm and a very strict confinement, leading the university system to become virtual online teaching. Taking into account the emotional deficiencies originated during the pandemic, among the most powerful tools to achieve engagement along with the identification, control and management of emotions is emotional intelligence (EI). The present study aims to establish the effect of the current confinement on the teaching-learning process and academic performance and the impact of the application of EI on university students. In total, 47 volunteers of the second course of the Degree in Pharmacy of the University of Granada (Spain) took part in this experience. Two temporary periods were established: at the beginning of the confinement period and after teaching several concepts of emotional intelligence online for two months. The Maslach Burnout Inventory-Student Survey Inventory (MBI-SS) and the Spanish version of Utrech Work Engagement Scale-Students (UWES-S) were used to evaluate the intervention. In total, 63.5% of the students presented academic burnout during the confinement before the intervention. After the EI workshops and seminars, only 31.1% presented academic burnout. Before the intervention with the emotional intelligence workshops, 44.6% experienced exhaustion, 41.7% cynicism and 60.3% felt it was ineffective in their academic performance. After the emotional intelligence workshops and seminars, 29.1% experienced exhaustion, 30.1% cynicism and 28.8% felt it was ineffective. The scores achieved after the study of EI in physiology classes led to better levels in all the variables studied. Students managed their adaptive processes more adequately and regulated their emotions better, as they felt less academic burnout and more engaged in their academic activities at the end of the study of EI through physiology.

## 1. Introduction

The recent appearance and rapid spread of the new SARS-CoV-2 coronavirus meant taking unprecedented measures to control the pandemic, which in Spain reached a health pressure that forced a state of alarm and a very strict confinement, which forced the university system to become virtual online teaching. The unexpected and drastic change in the world paradigm affected the educational system in a particular way. Confinement creates significant risks to the health and well-being of university students. It is common for young people in quarantine periods to show high levels of stress, anxiety, confusion and anger [1].

During confinement, the two factors that most affect physical and psychological well-being are the loss of habits and routines and psychosocial stress, according to the first study that analyzes the psychological impact of quarantine in China [2]. The interruption of habits during confinement and the establishment of unhealthy ones (bad eating habits, irregular sleep patterns, sedentary lifestyle and increased use of digital devices) can lead to health problems. The conditions that accompany a pandemic include various sources of stress for people. Studies on stressful situations and emergencies make it possible to summarize the main variables involved in the psychological impact, such as the following: fear of virus infection and diseases, the manifestation of feelings of frustration and boredom, not being able to meet basic needs and not having of information and clear guidelines for action [1] or the presence of previous mental health problems or financial problems [3]. Additionally, stigma and social rejection in the case of people infected or exposed to the disease can be a trigger for a worse adaptation [1]. All these conditions can profoundly influence academic performance in the students, and therefore, training, learning and academic achievement can be seriously impaired and could trigger sensations of exhaustion, negative attitude of self-criticism, and loss of interest (cynicism).

In the academic context, the effects of confinement can be measured through academic burnout, which is a variable used to evaluate psychological well-being related to studies [4]. This burnout is understood as a consequence and response to chronic stress linked to the role, activity and academic context of a malignant, insidious nature which can affect the development, commitment and satisfaction of students with their training and academic life, in addition to their psychosocial health [5]. The latest trends in burnout research have turned towards the study of its opposite trend: engagement in university students [6]. This change can be considered an effect of the rise in recent years of the so-called ”Positive Psychology”, which is concerned with the proper functioning and strengths of the individual in the face of negative events, thus trying to overcome the approaches centered on the deficit and pathology highly widespread within the field of psychology [7].

Engagement is defined as a positive mental state related to studies and characterized by vigor in the tasks as a student, high levels of dedication to studies and, finally, absorption in the aforementioned tasks [6]. Taking into account the emotional deficiencies originated during the pandemic, among the most powerful tools to achieve engagement along with the identification, control and management of emotions is emotional intelligence (EI). This refers to the ability to control emotions, own feelings and others’ feelings with the intention of guiding thoughts and actions, allowing the effective regulation of emotion in oneself and in others, and the use of feelings to motivate, plan and succeed in life [8].

Because confinement creates significant risks to the health and well-being of the university students, showing high levels of stress, anxiety and academic burnout, we decided to improve their academic engagement along with the identification, control and management of emotions by teaching some EI workshops. Taking into account these antecedents, the present study aims to establish the effect of the current confinement on the academic burnout and engagement, together with the impact of the application of emotional intelligence on university students.

## 2. Materials and Methods

### 2.1. Population and Intervention

Before the experience, the university students that wanted to take part in this educational strategy were selected. This study was approved by the University of Granada ethical committee (0060-N-18). In total, 47 volunteers of the second course of the Degree in Pharmacy of the University of Granada (Spain) took part in this experience. From this moment, two temporary periods were established: at the beginning of the confinement period and after teaching several concepts of EI online for two months. The topics reviewed in the seminars and workshops were:

- General physiological concepts linked to EI:
  ◦ Introductory activity: Integration dynamics.
  ◦ Learning with a neuroscientific approach.
  ◦ Brain organization (central nervous system, peripheral and autonomous).
  ◦ Neural information processing (synapse).
  ◦ Brain chemistry, hormones and behavior (neurotransmitters, importance of hormones and neuroendocrine integration).
- Brain and learning:
  ◦ Anatomy and brain physiology.
  ◦ Functions of the cerebral hemispheres.
- Emotions and learning:
  ◦ The emotional brain (limbic system and the role of the amygdala).
  ◦ Recommendations and exercises for brain care.
- EI development:
  ◦ Definition and evolution of the term intelligence.
  ◦ Definition of EI.

Basic components of EI and emotional competences:

These are the set of knowledge, abilities, skills and attitudes necessary to become aware of, understand, express and regulate emotional phenomena appropriately. The purpose of these competencies is aimed at providing added value to professional functions and promoting personal and social well-being. The emotional competencies that were worked on in the workshops were structured in five blocks: emotional self-knowledge, self-control, motivation, empathy and social skills. Emotional self-knowledge refers to the degree of knowledge one has of one’s own emotions and of the emotions of others, understanding how they can affect them, what produces them and having the ability to speak about them. Self-control means having the ability to express an appropriate response to the different situations that arise based on our emotional resources, our tolerance for certain stimuli and our independence of action. Motivation means being able to act with determination and enthusiasm. Empathy is a set of competencies that facilitate interpersonal relationships and promote group work, cohesion and team spirit. Social skills are a set of skills, attitudes and values that promote the construction of personal and social well-being.

Two questionnaires containing the dimensions of academic burnout and engagement were carried out compared to the studies before and after the interventions with the EI workshops. Code numbers instead of names were assigned to each completed questionnaire in order to maintain confidentiality.

### 2.2. Instruments

The Maslach Burnout Inventory-Student Survey Inventory (MBI-SS) [6] was used—an inventory adapted from the translation of the Maslach Burnout Inventory (MBI-GS) instrument, prepared by Maslach and Jackson [9]. The instrument assesses the dimensions of emotional exhaustion and cynicism through 11 items. (1) Emotional exhaustion: assesses the experience or feeling of being physically, mentally and emotionally exhausted, and with a feeling of not being able to perform some academic activities. (2) Cynicism: it evaluates the negative attitude of the student towards their studies, evidenced by self-criticism, devaluation, loss of interest and importance and value towards the study. (3) Academic self-efficacy: assesses the student’s perception of the competence in their studies.

We also used the Spanish version of Utrech Work Engagement Scale-Students (UWES-S) by Schaufeli, et al. [6], which includes different dimensions of student work. (1) Vigor: it evaluates energy and mental resistance while studying, the desire to invest effort, time, persistence in the study, even when obstacles and barriers appear, through six items. (2) Dedication: assesses the sense of meaning, enthusiasm, inspiration, pride and challenge related to studies. (3) Absorption: evaluates a pleasant state of total immersion in work, where the individual is unable to separate from work despite the fact that a long time has passed. All items score on a 7-point frequency scale, ranging from 0 (never) to 6 (always).

To evaluate the syndromes, the items of exhaustion and cynicism (academic burnout) and of dedication, absorption and vigor (academic engagement) were surveyed and, later, the scores were located according to the categories of low, medium low, medium high and high.

### 2.3. Statistical Analysis

Descriptive parameters were performed through frequency and descriptive analysis. The Student’s *t* was used to assess the significant differences in the three aspects of academic burnout and the three academic engagement factors before and after the intervention with the workshops. In addition, Spearman’s correlation coefficient was carried out to assess the relationship between the subscales of the academic burnout and engagement of the participants. The SPSS version 24.0 (SPSS Statistics for Windows, SPSS INC., Chicago, IL, USA) software was used for data analysis.

## 3. Results

In total, 47 volunteers of the second course of the Degree in Pharmacy of the University of Granada (Spain) took part in this experience. Table 1 shows the demographic variables of the subjects.

The results show that 63.5% of the students featured academic burnout (distributed in high and medium high levels) during the confinement before the intervention with the EI workshops, which suggests that the students experience the feeling of not being capable of achieving good academic results and a cynical attitude about the value and meaning of work. After the EI workshops and seminars, only 31.1% presented academic burnout (high and medium high levels). 

Before the intervention with the EI workshops, 44.6% experienced exhaustion, 41.7% cynicism and 60.3% felt it was ineffective in their academic performance. After the EI workshops and seminars, 29.1% experienced exhaustion, 30.1% cynicism and 28.8% felt it was ineffective. The presence of academic engagement was found in 39% of university students (distributed in medium high and high levels) before the intervention and in 57% after the intervention with workshops and seminars, showing the positive influence of the intervention on academic performance. 

The internal consistency (Cronbach’s alpha) of the instruments, was as follows: emotional exhaustion (α = 0.77), cynicism (α = 0.72), academic self-efficacy (α = 0.73), vigor (α = 0.71), dedication (α = 0.72) and absorption (α = 0.75). Table 2 shows the different scores used in the study before and after the intervention.

Before the workshops, emotional exhaustion correlated positively with cynicism (0.381, *p* < 0.01), and negatively with academic self-efficacy (–0.351, *p* < 0.01) and with satisfaction with studies (–0.298, *p* < 0.01). A positive correlation was also found between exhaustion and cynicism (0.381, *p* < 0.01), and a negative relationship was found between exhaustion and academic self-efficacy (–0.389, *p* < 0.01) and between cynicism and academic self-efficacy (–0.295, *p* < 0.01). A negative correlation was also found between cynicism and self-efficacy (–0.395, *p* < 0.01) and cynicism and satisfaction (–0.321, *p* < 0.01). 

After the workshops, emotional exhaustion correlated positively with cynicism (0.201, *p* < 0.05), and negatively with academic self-efficacy (–0.205, *p* < 0.05) and with satisfaction with studies (–0.221, *p* < 0.01). A positive correlation was also found between exhaustion and cynicism (0.285, *p* < 0.01), and a negative relationship was found between exhaustion and academic self-efficacy (–0.225, *p* < 0.01) and between cynicism and academic self-efficacy (–0.201, *p* < 0.05). A negative correlation was also found between cynicism and self-efficacy (–0.276, *p* < 0.01) and cynicism and satisfaction (–0.265, *p* < 0.01). 

Before the workshops, a positive correlation was found between vigor and dedication (0.380, *p* < 0.01), vigor and absorption (0.315, *p* < 0.01), vigor and satisfaction with studies (0.297, *p* < 0.01), academic vigor and self-efficacy (0.381, *p* < 0.01), vigor and dedication (0.265, *p* < 0.05) and vigor and absorption (0.290, *p* < 0.01). Exhaustion was negatively correlated with vigor (–0.435, *p* < 0.01), as well as cynicism with vigor (–0.277, *p* < 0.01) and cynicism with dedication (–0.285, *p* < 0.01). A positive correlation was also observed between exhaustion and cynicism (0.421, *p* < 0.001), and vigor was positively correlated with dedication (0.373, *p* < 0.01) and absorption (0.329, *p* < 0.01).

After the workshops, a positive correlation was found between vigor and dedication (0.525, *p* < 0.001), vigor and absorption (0.435, *p* < 0.01), vigor and satisfaction with studies (0.479, *p* < 0.001), academic vigor and self-efficacy (0.521, *p* < 0.001), vigor and dedication (0.483, *p* < 0.001) and vigor and absorption (0.402, *p* < 0.01). Exhaustion was negatively correlated with vigor (–0.283, *p* < 0.01), as well as cynicism with vigor (–0.239, *p* < 0.01) and cynicism with dedication (–0.301, *p* < 0.01). A positive correlation was also observed between exhaustion and cynicism (0.322, *p* < 0.01), and vigor was positively correlated with dedication (0.422, *p* < 0.01) and absorption (0.420, *p* < 0.01).

## 4. Discussion

This research aimed to describe the impact of various EI workshops and seminars on academic burnout and engagement of university students from the Faculty of Pharmacy (University of Granada) during the lockdown and confinement caused by COVID-19.

According to Goleman [10], it is essential to be aware of the own emotions, to know what a person feels and to have a positive and objective self-concept as well as of others. EI is understood as a set of skills that serve to express and control feelings in the most appropriate way in the personal and social field, including a good management of feelings, motivation, perseverance, empathy and of mental agility. In stressful situations, as we have been able to verify in the surveyed students, EI has helped students to interact with the world in a receptive and appropriate way to obtain an optimal adaptation to the learning process.

The tripartite model of EI postulates three levels of the concept: the knowledge level refers to the complexity and width of emotion knowledge; the ability level refers to the ability to apply emotion knowledge in an emotional situation and to implement a given strategy and finally; and the trait level refers to emotion-related dispositions, namely, the propensity to behave in a certain way in emotional situations. However, these three levels of EI are poorly connected: knowledge does not always translate into abilities, which, in turn, do not always translate into usual behavior [11,12]. Therefore, we can affirm that a longer intervention would be desirable to translate the knowledge of EI into usual behavior, especially after the confinement. 

Cognition and emotion are two aspects that exist together and have become inextricably linked in the mind of the individual, which leads them to act together, linked to the knowledge acquired. Stressful or unexpected situations, as in the case of confinement due to the pandemic, had a negative influence on the academic engagement and burnout of students. In fact, it is thanks to the need or interest that a person presents to acquire certain knowledge that emotions and feelings influence its acquisition; hence, we can affirm that our students improved their engagement and reduce their burnout once they knew several concepts of EI. This relationship between reason and emotion makes it possible to generate the adaptive capacity of the person, whose concrete manifestation is seen in the power to give answers and solutions, effectively, to the problems that arise linked to interpersonal relationships and disruptive behaviors, favoring a change in psychological well-being and academic performance [13].

In addition to the possible negative psychological effects due to the conditions of the confinement, the characteristics of the pandemic and the multiple associated factors, the lockdown can be perceived as an adversity of high psychosocial stress. Taking into account the results obtained in the current study, we can affirm that it would be important to include emotional education in the university to improve the teaching-learning process. It should be implemented gradually due to its complex structure. In addition, the study of EI allows the student to have more possibilities to adapt to the different situations and to obtain success in the projects proposed [14].

There is a range of reasons postulating that adequate indices of EI can help to solve academic and daily problems more successfully [15], obtaining a remarkable degree of psychological well–being [14,16]. In this sense, a good degree of EI can help people to collaborate more with the people around them, a fundamental skill in university teaching [17]. Poor levels of EI have also been shown to serve as predictors of stress development and the learning process [18].

Emotion favors attention, memory and learning, acting as booster for learning and development, but also an obstacle or a brake in case of negative feelings. Affectivity is a component of learning, and neuroscience studies are providing scientific support to the uniqueness of the person to the interaction between what we feel, what we think and how we act. Other research highlights that the role of emotions in learning is crucial [19]. Both emotion and feeling can promote learning by intensifying the activity of neural networks and thus strengthening synaptic connections.

### Limitations and Future Perspectives

It is important to continue studying academic burnout and engagement to correlate them with other psychological variables such as depression or other disorders, which will allow an understanding of the attitude of students to academic performance. Likewise, it would be necessary to study these variables differentiating by gender, increasing the population of the study and in different faculties and academic degrees to find indices that allow contrasting these results. Additionally, a longer intervention would be desirable to translate the knowledge of EI into usual behavior, especially after the confinement. It is also important to design programs aimed at strengthening teaching-learning processes, as well as intervention actions aimed at reducing burnout, especially in students with lower academic results.

## 5. Conclusions

A high rate of students featured academic burnout during the confinement before the intervention with the EI workshops. The study of several concepts of emotional intelligence in physiology classes led to better regulation of the students’ emotions; therefore, they felt less academic burnout and more engaged in their academic activities—facts that could improve the teaching–learning process during the confinement period and lockdown. 

## Figures and Tables

**Table 1:** Demographic variables of the subjects.

	Male	Female
Age (years)	20 ± 2.1	20 ± 1.8
Number of subjects	19	28
% of population	40.42	59.58

**Table 2:** Mean of scales used in the study (data presented as mean ± standard deviation).

	Before Intervention	After Intervention	Student’s *t* Test	Cronbach’s Alpha
MBI-SS (academic burnout)				
Emotional exhaustion	5.26 ± 1.22	4.31 ± 1.11	*p* < 0.01	0.77
Cynicism	3.11 ± 1.08	2.31 ± 1.39	*p* < 0.01	0.72
Academic self-efficacy	3.15 ± 1.26	4.63 ± 1.14	*p* < 0.001	0.73
UWES-S (academic engagement)				
Vigor	2.52 ± 1.22	3.76 ± 1.17	*p* < 0.01	0.71
Dedication	3.42 ± 1.42	5.17 ± 1.29	*p* < 0.001	0.72
Absorption	3.11 ± 1.11	4.17 ± 1.33	*p* < 0.01	0.75
